# Immunomodulatory therapies in community-acquired pneumonia: a protocol for a systematic review and network meta-analysis

**DOI:** 10.1136/bmjopen-2025-098994

**Published:** 2025-07-10

**Authors:** Luke Flower, Joseph Newman, Dominique-Laurent Couturier, Martin Law, Charlotte Summers

**Affiliations:** 1 Victor Phillip Dahdaleh Heart & Lung Research Institute, University of Cambridge, Cambridge, UK; 2 Cambridge University Hospitals NHS Foundation Trust, Cambridge, UK; 3 Royal Papworth Hospital NHS Foundation Trust, Cambridge, UK; 4 University of Cambridge, MRC Biostatistics Unit, Cambridge, UK; 5 Papworth Trial Unit Collaboration, Royal Papworth Hospital, Cambridge, UK

**Keywords:** Systematic Review, Meta-Analysis, INFECTIOUS DISEASES, RESPIRATORY MEDICINE (see Thoracic Medicine), INTENSIVE & CRITICAL CARE

## Abstract

**Abstract:**

**Introduction:**

Community-acquired pneumonia is the leading global cause of infection-related death. A subset of patients with pneumonia develops aberrant immune responses, resulting in harmful inflammation, tissue damage and significant mortality. Immunomodulatory therapies aim to blunt this dysregulated immune response and reduce resultant injury. No consensus exists on the use or impacts of immunomodulatory therapies in the management of community-acquired pneumonia. This protocol describes the methods we will use to undertake a systematic review and network meta-analysis of the effects of immunomodulatory therapies on the mortality of patients with community-acquired pneumonia.

**Methods:**

We will undertake a systematic review and network meta-analysis investigating the use of immunomodulatory therapies in community-acquired pneumonia. Our protocol has been developed and reported following the Preferred Reporting Items for Systematic Reviews and Meta-Analyses Protocols guidelines and prospectively registered with PROSPERO (CRD42024565301). The primary objectives of this work are to compare the impact of immunomodulatory therapies on 28-day and 90-day mortality in adult patients admitted to hospital with a primary diagnosis of community-acquired pneumonia. The secondary objectives of this work are to identify any differences in the effectiveness of these immunomodulatory therapies in managing community-acquired pneumonia of differing aetiology and severity.

We will conduct a literature search of Medline, Embase, Scopus, Web of Science and Global Health for all relevant articles until 30 June 2024. All observational, interventional and epidemiological studies published in English will be included, and each type of study design will be examined separately. All studies will have their titles and abstracts independently screened by two reviewers, followed by a full article eligibility review and data extraction. A third reviewer will adjudicate any disagreements. Data extracted will include, but not be limited to, the study design, country in which it was undertaken, patient characteristics (eg, age, sex, cause of CAP, severity of CAP), details regarding the immunomodulatory therapy and dosing used and the 28-day and 90-day mortality of each study arm.

**Analysis:**

The risk of bias will be assessed using the Risk of Bias in Non-randomised Studies - of Exposure tool for non-randomised studies and the Cochrane Risk of Bias 2 tool for randomised control trials. The quality of evidence will be evaluated using the Grading of Recommendations, Assessment, Development, and Evaluations for network meta-analysis framework. A quantitative synthesis of data is planned for 28-day and 90-day mortality rates.

We will fit a random-effects network meta-analysis model that includes random effects for between-study heterogeneity and for inconsistency. This will be done using the metafor package for R. We will use a contrast-based approach, modelling estimated treatment effects using reference treatments. In the case of the primary objective, this will be the log odds ratio (OR) of mortality in one treatment compared with another.

Each type of study design will be examined separately. Treatments using the same immunotherapy at different doses may be grouped if appropriate.

**Ethical approval and dissemination:**

This will be a systematic review of published literature; therefore, ethical approval is not required. To ensure communication of our findings, we will publish our results in a peer-reviewed journal and present our findings at appropriate local, national and international meetings.

**PROSPERO registration number:**

CRD42024565301.

**STRENGTHS AND LIMITATIONS OF THIS STUDY**

- The comprehensive search terms described are a notable strength of the study, increasing the probability of synthesising a reliable result of clinical relevance.
- A robust statistical approach is proposed for our network meta-analysis, ensuring accurate and reliable results by accounting for variability across studies.
- An inherent limitation is the possibility of heterogeneity of patient cohorts, drug choice and dosing between included studies.
- Including a wide variety of treatments and doses may result in inconsistent results.

## Introduction

 Community-acquired pneumonia (CAP) is the leading global cause of infection-related death, accounting for >2.5 million deaths each year.^1 2^ CAP may present with a range of clinical phenotypes, from mild infection requiring no intervention to multi-organ failure necessitating invasive treatments.^1^ Approximately 11% of patients admitted to hospital with CAP require admission to critical care, with an associated mortality of 50%.^3 4^

The spectrum of disease severity seen in CAP is partly due to variations in the host immune response.^5 6^ While the initial immune response to infection may be beneficial, some patients develop an aberrant inflammatory response, resulting in the excess release of inflammatory mediators and activation of deleterious downstream immune cell functions (eg, reactive oxygen species production).^7^,^9^ One consequence of this pro-inflammatory response can be acute respiratory distress syndrome (ARDS), which results in damage to lung tissue, pulmonary oedema, refractory hypoxaemia and death in up to 50% of patients.^7 8 10^

Immunomodulatory therapies dampen the dysregulated immune response, reducing disease severity and associated mortality. The COVID-19 pandemic highlights the pivotal role immunomodulatory therapies can play in infectious disease.^611^,^17^ A range of immunomodulatory therapies exist, with some acting via broad anti-inflammatory mechanisms (ie, corticosteroids) and others targeting specific inflammatory pathways, mediators or receptors (ie, the IL-1 receptor antagonist Anakinra).^13 18^

Despite the increasing use of immunomodulatory therapies in CAP, little consensus exists regarding their effects on mortality or the superiority of one drug over another. Current international guidelines recommend using corticosteroids in severe CAP with associated shock or ARDS, supported by Dequin *et al* demonstrating a mortality benefit in this cohort.^716 19^,^21^ However, the UK’s National Institute for Healthcare and Clinical Excellence does not recommend the routine use of corticosteroids in hospitalised patients with CAP.^720^,^22^ There are few guidelines regarding using other immunomodulatory therapies, with those that exist constrained to specific scenarios (eg, Tociluzimab in severe COVID-19).^17^

A preliminary search of PROSPERO, MEDLINE and the Cochrane Database of Systematic Reviews also identified no current systematic reviews being undertaken on the topic.^11 18 23^ Therefore, a systematic review and network meta-analysis are required to consolidate the current published evidence surrounding the use of immunomodulatory therapy in patients admitted to the hospital with CAP. Such work would represent a step forward in managing the disease and help guide clinical decision-making, guideline development and future research.

## Methods

We will undertake a systematic review and network meta-analysis investigating the use of immunomodulatory therapies in adults hospitalised with community-acquired pneumonia. This systematic review and network meta-analysis has been prospectively registered with PROSPERO (PROSPERO CRD42024565301), thus reducing the risk of duplication and improving transparency in the review process. This protocol has been prepared using the Preferred Reporting Items for Systematic Reviews and Meta-Analyses Protocols (PRISMA-P) guidelines.

### Objectives

The principal objective of this systematic review is to evaluate the effect of immunomodulatory therapies on the 28-day and 90-day mortality of adult patients admitted to hospital with CAP. We will also discern any differential mortality effects observed between CAP of different aetiologies (eg, bacterial vs viral) and between patients with differing degrees of disease severity.

### Eligibility criteria

#### Type of studies

This systematic review will include all observational, interventional and epidemiological studies involving adult patients hospitalised due to CAP, including hospital ward and critical care admissions. Only studies published in English will be included. Narrative reviews and non-English transcripts will be excluded.

#### Type of participants

We will consider all studies investigating immunomodulatory therapies in the management of adult (aged≥18 years) patients hospitalised with a primary diagnosis of CAP. We will accept a diagnosis of CAP based on national or international guidelines (eg, defined by the American Thoracic Society and NICE as pneumonia contracted outside of the healthcare system) or clinical judgement.^24 25^

#### Types of interventions

We will include all studies investigating the use of immunomodulatory therapies in the management of adults hospitalised with CAP, including studies that compare different immunomodulatory therapies to the standard of care or different immunomodulatory therapies to each other. A network meta-analysis will assess differential effects between immunomodulatory therapies across studies. The list of interventions searched will include all immunomodulation therapies known to affect immune pathways relevant to CAP, including passive immunotherapies (eg, steroids). The final list of immunomodulatory therapies was informed by Drugbank (accessed 01/07/2024), Pubmed/Medline searches and systematic reviews investigating their use in other disease states (ie, sepsis).^26^

#### Types of outcomes

This review will calculate the odds ratio (OR) of all-cause mortality at 28 days and 90 days for patients hospitalised with a primary diagnosis of CAP. Mortality is an important and widely recognised measure of intervention effectiveness in infectious diseases. It is pertinent for this patient cohort, as complications of CAP, such as ARDS, are associated with mortality rates exceeding 50%.^7 10^ The 28-day and 90-day mortality time points selected are commonly used in clinical trials and will provide insights into the short-term and longer-term effectiveness of immunomodulatory therapy in hospitalised adults with CAP. We intend to estimate mortality in a range of settings:

- Observational vs interventional studies
- Randomised control trials vs other designs
- Differing endpoints: 28 days vs 90 days
- The region or continent that the study was conducted in
- The type of immunomodulatory therapy used
- Dose of immunomodulatory therapy used
- Study standard of care (in the event of this differing across studies)
- The severity of CAP (eg, those requiring critical care admission)
- The aetiology of CAP (eg, bacterial vs viral vs fungal)

#### Patient and public involvement

The James Lind Alliance Priority Setting Partnership for Intensive Care findings were used in conceptualising this work, with infections and respiratory complications identified as one of the top research priorities by patients, families, doctors, nurses, and allied healthcare professionals.^27^

### Search strategy

We will search Medline, Embase, Scopus, Web of Science and Global Health for all relevant articles published from inception until 30 June 2024. We anticipate completing the analysis by July 2025. Boolean logic operators (ie, AND, OR and NOT), phrase searching, truncation tools and wildcards will be used to optimise the sensitivity and specificity of the search. Fields searched will include the article title, abstract and MeSH terms. Search terms were developed by identifying similar reviews investigating other conditions associated with aberrant inflammation (ie, sepsis), preliminary test searches and consultation with an experienced university librarian and subject matter experts. A preliminary test of the search strategy will be performed to assess its effectiveness and determine any modification requirements. Following this, a full search will be undertaken with the assistance of an experienced university librarian. The full example search strategy, formatted for the Medline database, is available in online supplemental file 1. This strategy will be adapted for use in alternative databases.

#### Study selection

Records from the electronic databases searched will be deduplicated and imported into Covidence.^28^ Two individuals will independently screen each paper against the pre-defined inclusion and exclusion criteria and will be blinded to each other’s decision. Where a consensus cannot be reached, disagreements will be adjudicated by a third reviewer.

### Data management

#### Data extraction

Screening will be undertaken using the Covidence systematic review management tool, and relevant data will then be extracted into a pre-designed Excel template for analysis.^27^ Data to be extracted will include:

1. Data regarding the publication and design of each study: study title, authorship, year of publication, funding source, country in which it was conducted, study design and eligibility criteria.
2. Patient characteristics: age, sex, ethnicity, co-morbidity, cause of CAP (eg, bacterial vs viral), severity of CAP and number of patients included.
3. Details regarding the intervention: immunomodulatory therapy used, dosage, timing of initiation relative to CAP diagnosis, duration of treatment and standard of care.
4. Study details: The design and methodology of the studies.
5. Patient outcome: 28-day and 90-day all-cause mortality for each trial arm.
6. One researcher will extract and record data in Excel, and another will review the extraction. A third reviewer will resolve any disagreements.

#### Risk of bias in individual studies

For non-randomised studies, the risk of bias will be assessed using the Risk Of Bias In Non-randomised Studies - of Exposure (ROBINS-E) tool and stratify studies as low risk, some concerns, high risk, very high risk or unable to ascertain. For randomised studies, the Cochrane Risk of Bias 2 tool will be used. The risk of bias scores will be checked using random study selection by an independent reviewer. The quality of evidence will be assessed using the Grading of Recommendations, Assessment, Development and Evaluations (GRADE) for network meta-analysis framework, encompassing risk of bias, imprecision, inconsistency, indirectness and publication bias.^29^ Sensitivity analyses will be undertaken that exclude studies with a high risk of bias.

#### Data synthesis

A quantitative data synthesis is planned for 28-day and 90-day mortality rates. Data will be analysed using R version 4.5.0 or newer and R package metafor.^30 31^ A random-effects network meta-analysis model that includes random effects for between-study heterogeneity and for inconsistency will be used.^32 33^ We will use a contrast-based approach, modelling estimated treatment effects using reference treatments. For the primary objective, this will involve estimating the log OR of mortality for one treatment compared with another. The OR of mortality will be presented with corresponding 95% confidence intervals, with the weighting of each study demonstrated by the square size within forest plots.

Heterogeneity between the cohorts will be assessed by Cochran’s Q (χ²) and quantified by I² with >50% taken as significant heterogeneity. We will present the findings narratively where pooling is not feasible due to a lack of data or heterogeneity. Sensitivity analysis will be undertaken to assess the effects of removing studies judged to have high, very high or unknown risk of bias. Publication bias will be assessed using a funnel plot and Egger’s test, with significance taken as p<0.05.

#### Subgroup analyses

Subgroup analyses will be performed to examine potential heterogeneity in treatment effect by aetiology, where sufficient data are available, as underlying cause may influence both disease progression and response to treatment. Subgroups will include:

- COVID-19
- Non-COVID-19 viral infections
- Bacterial infections
- Fungal infections
- No identified infectious cause

Furthermore, subgroup analyses will also be conducted to assess differences between drugs with distinct mechanisms of action, as this may reveal clinically meaningful variations in efficacy based on pharmacological differences. Proposed subgroups will include:

- Cytokine inhibitors stratified by cytokine (eg, IL-1, IL-6, IL-33)
- Corticosteroids stratified by steroid (eg, dexamethasone, hydrocortisone, prednisolone)
- Immunoglobulins
- JAK inhibitors
- G-CSF/GM-CSF
- GM-CSF inhibitors
- Plasma

A network meta-analysis (and corresponding diagram) may be presented that ‘lumps’ the treatments in the above way, if appropriate. Finally, a subgroup analysis based on disease severity, specifically critically ill vs non-critically ill patients, will be performed, as treatment effects may differ significantly depending on baseline severity. The results of all subgroup analyses will be discussed in relation to the results of the main analysis. The subgroup analyses are secondary analyses: they are for the purpose of hypothesis generation only, and we acknowledge our use of multiple testing.

### Amendments

Any protocol amendments will be documented with the date, description of the change and rationale, and will be reported in the final manuscript to ensure transparency.

## Discussion

To our knowledge, this will be the first comprehensive systematic review and network meta-analysis of immunomodulatory therapies in hospitalised adults with CAP. Community-acquired pneumonia is the leading global cause of infection-related death, and effective immunomodulatory therapies represent an area of unmet scientific need. Collating the findings of published studies will improve clinicians' and researchers’ understanding of the effects of immunomodulatory therapy on patient mortality, facilitate targeted research and improve treatment decision-making.

The strengths of this systematic review include the robust methodology and the comprehensive search terms proposed, which will permit the identification of relevant studies and increase the probability of reaching reliable conclusions.

### Limitations

An inherent limitation of a systematic review and meta-analysis of immunomodulatory therapy is the potential for heterogeneity of patient cohorts, drug use and outcome reporting. Furthermore, systematic reviews and meta-analyses may be affected by publication bias, where studies showing benefit from a therapy are more likely to be published than those that do not. We will assess for heterogeneity and publication and adjust our analyses and data presentation accordingly. A limitation of network meta-analysis in particular is the additional assumptions that result from combining direct and indirect evidence, including transitivity. To allow readers to empirically evaluate the assumption of transitivity, we will present tabulated data regarding participant and study characteristics by study. While limiting to English-language publications is a common criterion in large-scale systematic reviews and meta-analyses, we acknowledge the potential for selection bias. This decision is based on practical considerations, including the authors’ primary fluency in English, which may limit accurate assessment of non-English studies, as well as the need for consistency in reporting and methodology.

## Ethics and dissemination

This is a systematic review of published literature. Therefore, ethical approval is not required. We will ensure our findings are communicated with broad clinical and scientific communities by publishing our results in a peer-reviewed journal and presenting our findings at appropriate local, national and international meetings.

## Supplementary material

10.1136/bmjopen-2025-098994 online supplemental file 1
