# Supplementary material for: Immunomodulatory therapies in community-acquired pneumonia: a protocol for a systematic review and network meta-analysis
Source: BMJ Open. 2025 Jul 10;15(7):e098994. doi: 10.1136/bmjopen-2025-098994 (PMC12258272; doi:10.1136/bmjopen-2025-098994)
Supplement: online supplemental file 1 [file bmjopen-15-7-s001.docx]

**Supplementary material**

**Databases searched:**

- Medline (Ovid)
- Embase (Ovid)
- Scopus
- Web of Science
- Global Health (ebscohost)

**Fields searched:**

- Title
- Abstract
- MeSH terms

**Restrictions applied to searches:**

- English language
- All relevant publications to present

**Search terms:**

#1          ("adult*" or "over 18" or "over eighteen" or "18 and over" or "eighteen and over")

#2          Adult

#3          #1 or #2

#4          pneumonia

#5         “ acute respiratory distress syndrome” or ARDS or “respiratory distress syndrome” or “acute lung injury” or ALI

#6          #4 or #5

#7 Steroids or “adrenal cortex hormones”

#8 methylprednisolone or hydrocortisone or glucocorticoid* or dexamethasone or corticosteroid* or cortiso* or prednisolone

#9 #7 or #8

#10 immunomodulat* or (cytokine block*)

#11 (Risankizumab) or (Skyrizi) or (risankizumab-rzaa) or (BI-655066) or (ABBV-066) or (BI 655066) or (Tildrakizumab) or (Ilumya) or (Ilumetri) or (Tildrakizumab-asmn) or (IL-23p19 inhibitor) or (IgG1/k monoclonal antibod*) or (humanized IgG1/k monoclonal antibod*) or (MK-3222) or (SCH) or (SCH 900222) or (Guselkumab) or (Tremfya) or (Tremfya One-press) or (IL-23 inhibitor*) or (CNTO1959) or (Risankizumab) or (Skyrizi) or (risankizumab-rzaa) or (BI-655066) or (ABBV-066) or (BI 655066) or (Tildrakizumab) or (Ilumya) or (Ilumetri) or (Tildrakizumab-asmn) or (IL-23p19 inhibitor*) or (IgG1/k monoclonal antibod*) or (humani?ed IgG1/k monoclonal antibod*) or (MK-3222) or (SCH) or (SCH 900222) or (Guselkumab) or (Tremfya) or (Tremfya One-press) or (IL-23 inhibitor*) or (CNTO1959) or (Sirukumab) or (CNTO-136) or (Sarilumab) or (Kevzara) or (REGN88) or (SAR153191) or (Ixekizumab) or (Taltz) or (LY 2439821) or (LY-2439821) or (LY2439821) or (Siltuximab) or (Sylvant) or (CNTO 328) or (anti-IL-6 chimeric monoclonal antibod*) or (cCLB8) or (CLLB8) or (CNTO-328) or (CNTO328) or (Tocilizumab) or (Actemra) or (RoActemra) or (Actemra ACTpen) or (atlizumab) or (MRA) or (MSB-11456) or (MSB11456) or (R-1569) or (RG-1569) or (RHPM-1) or (RO-4877533) or (Secukinumab) or (Cosentyx) or (human IgG1κ monoclonal antibod*) or (AIN457) or (AIN457A) or (AIN-457) or (Brodalumab) or (Siliq) or (Kyntheum) or (KHK4827) or (AMG 827) or (KHK-4827) or (AMG-827) or (Briakinumab) or (ABT-874) or (Ustekinumab) or (Stelara) or (CNTO 1275) or (CNTO-1275) or (CNTO1275) or (TT 20) or (TT-20) or (TT20) or (Canakinumab) or (Ilaris) or (ACZ885) or (ACZ-885) or (Rilonacept) or (Arcalyst) or (Rolonacept regeneron) or (IL-1 Trap) or (interleukin-1 trap) or (Anakinra) or (Kineret) or (IL-1RA) or (Interleukin-1 receptor antagonist anakinra) or (Basiliximab) or (Simulect) or (chimeric CD25 monoclonal antibody) or (CHI-621) or (SDZ-CHI-621) or (Daclizumab) or (Zinbryta) or (Zenapax ) or (Dacliximab) or (Daclizumab beta) or (RO 24-7375) or (RO-24-7375) or (TNF-a inhibitors) or (Golimumab) or (Simponi) or (Simponi Aria) or (CNTO-148) or (CNTO 148) or (CNTO148) or (Certolizumab pegol ) or (Cimzia) or (CDP870) or (CDP 870 ) or (CDP-870) or (PHA-738144) or (Adalimumab) or (Humira) or (Mabura) or (Exemptia) or (Trudexa) or (Amgevita) or (Hyrimoz) or (Idacio) or (Imraldi) or (Amjevita) or (Cyltezo) or (Humira pen) or (Amsparity) or (Hadlima) or (Halimatoz) or (Hefiya) or (hulio) or ( yuflyma) or (solymbic) or (kromeya) or (adalimumab-adaz) or (adalimumab-adbm) or (adalimumab-afzb) or (adalimumab-atto) or (adalimumab-bwwd) or (adalimumab-fkjp) or (Adalimumab (genetical recombination)) or (ABP-501) or (BCD-057) or (BI-695501) or (BI695501) or (CHS-1420) or (D2E7) or (GP-2017) or (GP2017) or (LU-200134) or (LU200134) or (M-923) or (M923) or (MSB-11022) or (MSB11022) or (ONS-3010) or (SB-5) or (SB5) or (Afelimomab) or (Segard) or (MAK 195F) or (MAK-195F) or (LU-54107) or (LU54107) or (Infliximab) or (Remicade) or (Remsima) or (Inflectra) or (Infimab) or (Flixabi) or (Ixifi) or (Zessly) or (Avsola) or (Omvyence) or (Renflexis) or (infliximab-abda) or (infliximab-axxq) or (infliximab-dyyb) or (infliximab-qbtx) or (Infliximab (genetical recombination)) or (ABP 710) or (ABP-710) or (BOW-015) or (BOW015) or (CT-P-13) or (CT-P13) or (GP 1111) or (GP-1111) or (PF-06438179) or (TA-650) or (Etanercept) or (Enbrel) or (Benepali) or (Erelzi) or (Etacept) or (Benpali) or (Lifmior) or (Nepexto) or (Brenzys) or (etanercept-szzs) or (etanercept-ykro) or (Recombinant human TNF) or (rhu TNFR:Fc) or (rhu-TNFR:Fc) or (TNFR-immunoadhesin) or (CHS-0214) or (DWP-422) or (ENIA-11) or (GP-2015) or (GP2015) or (GP2015C) or (HD-203) or (HD203) or (LBEC-0101) or (LBEC0101) or (SB-4) or (SB4) or (cytofab) or (Recombinant human TNFa) or (anti-TNFa) or (Lenercept) or (recombinant TNF receptor p55) or (CDP571) or (Selective immunosuppressants) or (Upadacitinib ) or (Rinvoq) or (Upadacitinib hemihydrate) or (Upadacitinib tartrate) or (ABT-494) or (ABT 494) or (Baricitinib) or (Olumiant) or (Baricinix) or (Baricent) or (INCB28050) or (LY3009104) or (INCB028050) or (LY-3009104) or (Tofacitinib) or (Xeljanz) or (Jaquinus) or (Tofacinix) or (CP-690550) or (tasocitinib) or (CP-690,550) or (Tofacitinibum) or (CP 690550) or (CP- 690 550) or (CP-690 free base) or (CP-690-550) or (CP-690,550 free base) or (CP-690550 free base) or (CP690,550) or (CP690550) or (Ravulizumab ) or (Ultomiris) or (ALXN1210) or (ravulizumab-cwvz) or (ALXN-1210) or (Eculizumab) or (Soliris) or (Elizaria) or (5G1.1) or (H5G1-1) or (H5G1.1) or (H5G11) or (Siponimod) or (Mayzent) or (BAF-312) or (BAF312) or (NVP-BAF312-NX) or (Ozanimod) or (Zeposia) or (RPC-1063) or (Ozanimodum) or (RPC 1063) or (RPC1063) or (Fingolimod) or (Gilenya) or (Fingolimod accord) or (asn-fingolimod) or (Fingolimodum) or (FTY-720A) or (FTY720) or (Emapalumab) or (Gamifant) or (NI-0501) or (emapalumab-lzsg) or (Ropeginterferon alfa-2b) or (Besremi) or (AOP2014) or (Peginterferon alfa-2a, combinations) or (Peginterferon alfa-2b, combinations) or (Cepeginterferon alfa-2B) or ) or (Algeron) or (Cepeginterferon Alfa 2 B) or (Pegylated human interferon A-2B) or (Peginterferon beta-1a) or (Plegridy) or (Plegridy pen) or (PEG IFN-beta-1a) or (Polyethylene glycol-interferon beta-1a) or (BIIB-017) or (BIIB017) or (Albeterferon Alfa-2B) or (Albuferon) or (alb-IFN) or (Albinterferon) or (Albuferon alpha) or (Albumin-interferon alpha) or (Peginterferon alfa-2a) or (Pegasys) or (Pegylated Interferon alfa-2A) or (Pegylated interferon alfa-2a) or (Pegylated interferon alpha-2a) or (Pegylated-interferon alfa 2a) or (RO 25-8310/000) or (RO-25-8310/000) or (RO-258310000) or (Peginterferon alfa-2b) or (PegIntron) or (Sylatron) or (ViraferonPeg) or (PEG-Intron) or (Unitron Peg) or (Interferon alfacon-1) or (Advaferon) or (Infergen) or (IFN Alfacon-1) or (IFN-Con1) or or (Interferon beta-1b) or (Betaseron) or (Actoferon) or (Extavia) or (Betaferon) or (Interferon beta 1b (recombinant)) or (Interferon beta-1b,recombinant) or (Interferon-beta-1b) or (Recombinant interferon beta-1b) or (Interferon beta- 1a) or (Avonex) or (Rebif) or (CinnoVex) or (Rebif rebidose) or (interferon beta 1-alpha) or (Interferon beta 1-a) or (Interferon beta 1a) or (Interferon beta-1a (recombinant human)) or (Interferon beta-1a,recombinant) or (Interferon-beta-1a) or (SNG001) or (interferon alfa-n1) or (Wellferon) or (Interferon alpha-n1) or (Interferon alfa-2b) or (Intron-A) or (Introna) or (Locteron) or (Alpharona) or (IntronA) or (Realderon) or (Reaferon EC) or (Reaferon EC-lipint) or (Infagel) or (Recolin) or (Altevir) or (Kipferon) or (Giaferon) or (Opthalamoferon) or (Heberon Alfa R) or (Interferon alfa-2b, recombinant) or (Interferon alpha-2B) or (Interferon α-2b) or (Intron (Interferon α-2b)) or (r-INF-alpha) or (rIFN-alpha-2b) or (SCH 30500) or (SCH-30500) or (YM 14090) or (interferon alfa-2a) or (Roferon A) or (Interferon alfa-2a (genetical recombination)) or (Interferon alfa-2a (recombinant)) or (Interferon alfa-2a, recombinant) or (Interferon alfa-2a,recombinant) or (Interferon alpha-2a) or (Interferon-alfa-2a) or (Recombinant human interferon alfa-2a) or (Recombinant human interferon-alfa-2a) or (RO 22-8181) or (RO-22-8181) or (RO-228181) or (interferon gamma ) or (Actimmune) or (Immukin) or (Heberon gamma) or (IFNγ) or (Recombinant Interferon Gamma) or (Interferon beta natural ) or (interferon alfa natural ) or (Multiferon) or (HuIFN-alpha-Le) or (Colony stimulating factors) or (Pegteograstim) or (Empegfilgrastim) or (Colony-stimulating factor, granulocyte (synthetic human), 30-kilodalton pegylated) or (Balugrastim) or (Lipegfilgrastim) or (Lonquex) or (XM-22) or (XM22) or (Pegfilgrastim) or (Neulasta) or (Fulphila) or (Pelgraz) or (Lapelga) or (Pelmeg) or (Udenyca) or (Ziextenzo) or (Grasustek) or (Cegfila) or (Neupopeg) or (Nyvepria) or (Ristempa) or (Granulocyte colony-stimulating factor pegfilgrastim) or (peg-filgrastim) or (pegfilgrastim-apgf) or (pegfilgrastim-bmez) or (pegfilgrastim-cbqv) or (pegfilgrastim-jmdb) or (Ancestim) or (Stemgen) or (Lenograstim) or (Granocyte) or (Euprotin) or (Lenobio) or (Neutrogin) or (Lenograstim (genetical recombination)) or (Lenograstim rDNA) or (Sargramostim) or (Leukine) or (rGM-CSF) or (rHu GM-CSF) or (B1 61.012) or (B1-61012) or (Molgramostim) or (Filgrastim) or (Neupogen) or (Zarxio/Zarzio) or (Granix) or (Fraven) or (Nivestym) or (Tbo-filgrastim) or (Biocilin) or (Biofilgran) or (Endufil) or (Filatil) or (Filgen) or (Gran) or (Granulokine) or (Grimatin) or (Immunef ) or (jiexin) or (leucogen) or (leucostim) or (macroleuco) or (neukine) or (neutromax) or (neutroval) or (recombicyte) or (scilocyte) or (tevagastrim) or (white-c) or (accofil) or (biograstim) or (filgratim hexal) or (filgrastim ratiopharm) or (grastofil) or (nivestim) or (ratiograstim) or (tevagrastim) or (filgrastim-aafi) or (filgrastim-sndz) or (r-metHuG-CSF) or (recombinant methionyl human granulocyte colony stimulating factor) or (G-CSF) or (Granulocyte Colony Stimulating Factor) or (Tasonermin) or (Beromun) or (Tasonermina) or (TNF) or (Tumor necrosis factor, soluble form, nonglycosylated) or (Otilimab) or (GSK-3196165) or (GSK3196165) or (MOR-103) or (MOR103) or or (HuCAL antibody) or (r-hIL-7) or (human interleukin-7) or (recombinant interleukin-7) or (Glycosylated recombinant human interleukin-7) or (Interleukin 7 glycosylated) or (Interleukin-7 human recombinant) or (SH-polypeptide-42) or (CYT 107) or (CYT-107) or (Intravenous immunoglobulin) or (Asceniv) or (Bivigam) or (Gammagard) or (Gammaked) or (Gammaplex) or (Gamunex) or (Hizentra) or (Hyqvia) or (Igivnex) or (Octagam) or (Panzyga) or (Privigen) or (Xembify) or (Kiovig) or (Gamastan) or (Flebogamma) or (Cuvitru) or (Human immunoglobulin G) or (Human IGG) or (Human normal immunoglobulin) or (Immune globulin human) or (Immunoglobulin human) or (human immunoglobulin) or (Immunoglobulin G human) or (IVIg) or (Normal immunoglobulin human) or or (pentaglobin) or (IgM enriched immunoglobulins) or (IVIgGM) or (Adrecizumab) or (HAM 8101) or (HAM8101) or (tak-242) or (nangibotide) or (LR21)

Or (TLR4 antagnoist) or (Resatorvid) or (C1-esterase inhibitor) or (Cinryze) or (Berinert) or (Haegarda) or (human C1 inhibitor) or (C1 inhibitor human) or (C1-inhibiting factor) or (C1-inhibitor, plasma derived) or (Human C1-esterase inhibitor) or (Plasma protease C1 inhibitor) or (RVG-19303) or (bb-882)

#12 #9 or #10 or #11

#13 #3 and #6 and #12
