# Supplementary material for: Immunomodulatory therapies in community-acquired pneumonia: a protocol for a systematic review and network meta-analysis
Source: BMJ Open. 2025 Jul 10;15(7):e098994. doi: 10.1136/bmjopen-2025-098994 (PMC12258272; doi:10.1136/bmjopen-2025-098994)
Supplement: Supplementary file 2 [file bmjopen-2025-098994.reviewer_comments.pdf]

PEER REVIEW HISTORY

BMJ Open publishes all reviews undertaken for accepted manuscripts. Reviewers are asked to complete a checklist review form and are provided with free text boxes to elaborate on their assessment. These free text comments are reproduced below.

ARTICLE DETAILS

Title (Provisional)

Immunomodulatory therapies in community-acquired pneumonia: A protocol for a systematic review and network meta-analysis

Authors

Flower, Luke; Newman, Joseph; Couturier, Dominique; Law, Martin; Summers, Charlotte

---

VERSION 1 - REVIEW

**Reviewer**	**1**
**Name**	**Meyerowitz , Eric A.**
**Affiliation**	**Montefiore Medical Center**
**Date**	**11-Feb-2025**
**COI**	**None**

The authors are undertaking a worthy and important endeavor.

Overall, I think the protocol is extremely well thought out and reasonable. However, I think the authors should address specifically the potential importance of the specific etiology of CAP. I am glad to see they are planning a subgroup analysis - I would specifically suggest they consider adding some more subcategories that would allow them to more systematically address the question of whether and to what degree etiology matters. Some specific sub groups: "COVID" (where we know there is a benefit for people with hypoxemia), viral non-COVID, all viral (including COVID), bacterial (could divide into Strep pneumo and other), fungal (could specifically look at data for Pneumocystis, Coccidiomycosis, Histoplasmosis, etc), "no infectious etiology identified"

If the authors create well delineated categories, it will make their findings much more applicable to clinicians who need to make decisions about when and whether to use adjunctive corticosteroids (and specifically whether there are any forms of CAP where they should be avoided).

I look forward to seeing their findings!

**Reviewer**	**2**
**Name**	**Colunga Lozano, Luis Enrique**
**Affiliation**	**McMaster University, Department of Health Research Methods, Evidence, and Impact**
**Date**	**09-May-2025**
**COI**	**None**

This protocol outlines a systematic review and network meta-analysis designed to evaluate the effects of immunomodulatory therapies in adults hospitalized with community-acquired pneumonia. The primary objective is to assess the impact of these therapies on 28-day and 90-day mortality and to compare their effectiveness across different pneumonia etiologies and severities. The following recommendations aim to enhance the methodological rigour, clarity, and reliability of the proposed study:

1. Title Modification

I recommend removing the word “incorporating” from the title for clarity. The suggested title is:

“Immunomodulatory Therapies in Community-Acquired Pneumonia: A Protocol for a Systematic Review and Network Meta-Analysis”

2. Reporting Guidance

The protocol would benefit from including elements from the PRISMA-NMA extension. Specifically, please clarify how key assumptions of transitivity and inconherence will be assessed and addressed.

3. PROSPERO Registration

The PROSPERO registration (completed in July 2024) does not include the network meta-analysis component. To maintain consistency between the registered protocol and the current version, I recommend updating the PROSPERO record to reflect these methodological changes.

4. Introduction and Objective

On Page 7, Line 30, there is an inconsistency in the description of the type of meta-analysis. Please ensure consistent terminology is used throughout the document to specify that a network meta-analysis is planned.

5. Amendments

The PRISMA-P guideline includes a section dedicated to protocol amendments. I recommend including such a section to document any future changes and ensure full transparency.

6. Type of Studies

The eligibility criteria for included studies should be clarified. Non-randomised studies can be comparative or non-comparative, and interventional studies may have various designs (e.g., parallel-group, cluster-randomized, or cross-over). Please provide specific details on the study designs to be included and any that are excluded.

7. Language Restriction

The protocol states that only English-language studies will be included. As systematic reviews aim for comprehensive evidence synthesis, please provide a clear justification for this language restriction.

8. Subgroup Analyses

The authors plan multiple subgroup analyses to explore heterogeneity in effect estimates. I recommend creating a dedicated section to:

- List all planned subgroup analyses

- Justify their selection

- Describe how subgroup results will be integrated into the overall analysis

Additionally, I suggest using tools such as ICEMAN (Instrument to assess the Credibility of Effect Modification Analyses) to evaluate the credibility of subgroup effects:

Schandelmaier et al. CMAJ. 2020;192(32):E901-E906. doi: 10.1503/cmaj.200077

9. Search Strategy

On Page 12, Line 20, the inclusion of a "Search term summary" appears redundant, given that full search strategies are available in the supplementary materials. I suggest removing this summary from the main text.

10. Risk of Bias in Individual Studies

The authors plan to use ROBINS-E for non-randomized studies. However, if randomized controlled trials are also eligible, an appropriate tool should be used for RCTs. Please clarify this and consider using multiple tools where appropriate.

11. GRADE Framework

The current reference for the GRADE framework if for pairwise meta-analyses. For network meta-analyses, I recommend adopting a framework specifically designed for this context, such as:

GRADE for NMA (Izcovich et al., BMJ, 2023), or

CINeMA (Confidence in Network Meta-Analysis; Nikolakopoulou et al., PLoS Med, 2020)

Both approaches are aligned with GRADE principles but differ in complexity and implementation.

VERSION 1 - AUTHOR RESPONSE

Reviewer: 1
Eric A. Meyerowitz , Montefiore Medical Center
Comments to the Author:

The authors are undertaking a worthy and important endeavor.
Thank you for your kind words.

Overall, I think the protocol is extremely well thought out and reasonable.

Thank you - we're pleased to hear that you find the protocol well considered.

However, I think the authors should address specifically the potential importance of the specific etiology of CAP. I am glad to see they are planning a subgroup analysis - I would specifically suggest they consider adding some more subcategories that would allow them to more systematically address the question of whether and to what degree etiology matters. Some specific sub groups: "COVID" (where we know there is a benefit for people with hypoxemia), viral non-COVID, all viral (including COVID), bacterial (could divide into Strep pneumo and other), fungal (could specifically look at data for Pneumocystis, Coccidiomycosis, Histoplasmosis, etc), "no infectious etiology identified". If the authors create well delineated categories, it will make their findings much more applicable to clinicians who need to make decisions about when and whether to use adjunctive corticosteroids (and specifically whether there are any forms of CAP where they should be avoided).

Thank you for highlighting this important point. We agree subgroup analysis by CAP aetiology will be an important part of this work and have now updated the methods section to reflect this as follows:

"Subgroup analyses will examine possible heterogeneity of treatment effect regarding aetiology, where sufficient data are available. Subgroups will include:

- COVID-19
- Non-COVID-19 viral infections
- Bacterial infections
- Fungal infections
- No identified infectious cause

Subgroup analyses will also be conducted to assess differences between drugs with distinct mechanisms of action (e.g., IL-1 receptor antagonists vs. corticosteroids) and

across different study designs.”

I look forward to seeing their findings!

We look forward to sharing them!

Reviewer: 2
Dr. Luis Enrique Colunga Lozano, McMaster University
Comments to the Author:
This protocol outlines a systematic review and network meta-analysis designed to evaluate the effects of immunomodulatory therapies in adults hospitalized with community-acquired pneumonia. The primary objective is to assess the impact of these therapies on 28-day and 90-day mortality and to compare their effectiveness across different pneumonia etiologies and severities. The following recommendations aim to enhance the methodological rigour, clarity, and reliability of the proposed study:

1. Title Modification
I recommend removing the word "incorporating" from the title for clarity. The suggested title is:

"Immunomodulatory Therapies in Community-Acquired Pneumonia: A Protocol for a Systematic Review and Network Meta-Analysis"

Thank you for highlighting this, we have now adjusted the title as suggested to:

“Immunomodulatory Therapies in Community-Acquired Pneumonia: A Protocol for a Systematic Review and Network Meta-Analysis”

2. Reporting Guidance
The protocol would benefit from including elements from the PRISMA-NMA extension. Specifically, please clarify how key assumptions of transitivity and inconherence will be assessed and addressed.

We agree. We now include the possibility of transitivity as a limitation, and state that we will present participant and study characteristics in the paper. Incoherence/inconsistency is included in the network meta-analysis model, and we have now made this clearer.

“A limitation of network meta-analysis in particular are the additional assumptions that result from combining direct and indirect evidence, including transitivity. To allow readers to empirically evaluate the assumption of transitivity, we will present tabulated data regarding participant and study characteristics by study”.

3. PROSPERO Registration
The PROSPERO registration (completed in July 2024) does not include the network meta-analysis component. To maintain consistency between the registered protocol and the current version, I recommend updating the PROSPERO record to reflect these methodological changes.

Thank you for highlighting this, this has now been updated.

4. Introduction and Objective
On Page 7, Line 30, there is an inconsistency in the description of the type of meta-analysis. Please ensure consistent terminology is used throughout the document to specify that a network meta-analysis is planned.

Thank you for highlighting this. We have now corrected the text to ensure consistent use of terminology referring to a network meta-analysis throughout.

5. Amendments
The PRISMA-P guideline includes a section dedicated to protocol amendments. I recommend including such a section to document any future changes and ensure full transparency.

Thank you for highlighting this important point, we have now included such a section into the document as suggested:

"Any protocol amendments will be documented with the date, description of the change, and rationale, and will be reported in the final manuscript to ensure transparency."

6. Type of Studies
The eligibility criteria for included studies should be clarified. Non-randomised studies can be comparative or non-comparative, and interventional studies may have various designs (e.g., parallel-group, cluster-randomized, or cross-over). Please provide specific details on the study designs to be included and any that are excluded.

Thank you for highlighting this point. All study designs will be included in the analysis, including the various designs of interventional and non-randomised studies you have mentioned. We have highlighted this in the abstract and main body of the text as follows:

Abstract:

“All observational, interventional and epidemiological studies published in English will be included, and each type of study design will be examined separately.”

Main text:

“This systematic review will include all observational, interventional, and epidemiological studies involving adult patients hospitalised due to CAP, including hospital ward and critical care admissions.”

7. Language Restriction
The protocol states that only English-language studies will be included. As systematic reviews aim for comprehensive evidence synthesis, please provide a clear justification for this language restriction.

Whilst this is a common inclusion criteria in large scale systematic reviews and meta-analysis, we acknowledge its potential for selection bias. Specific reasons for this approach include practical considerations (the authorship is primarily English speaking which may limit our ability to accurately assess studies in other languages), alongside access to relevant literature, and consistency in reporting and methodology. An initial trial search of one database provided us with an estimate of >22,000 papers in the review, so we are confident that despite this exclusion criteria our review and meta-analysis will still be comprehensive. We have now acknowledged this in the manuscript:

“While limiting to English-language publications is a common criterion in large-scale systematic reviews and meta-analyses, we acknowledge the potential for selection bias. This decision is based on practical considerations, including the authors’ primary fluency in English, which may limit accurate assessment of non-English studies, as well as the need for consistency in reporting and methodology.”

8. Subgroup Analyses
The authors plan multiple subgroup analyses to explore heterogeneity in effect estimates. I recommend creating a dedicated section to:
- List all planned subgroup analyses
- Justify their selection
- Describe how subgroup results will be integrated into the overall analysis
Additionally, I suggest using tools such as ICEMAN (Instrument to assess the Credibility of Effect Modification Analyses) to evaluate the credibility of subgroup

effects: Schandelmaier et al. CMAJ. 2020;192(32):E901-E906. doi: 10.1503/cmaj.200077

Thank you for this important point. We have now created a dedicated section as suggested, outlining and justifying the proposed subgroup analyses, while acknowledging that these are somewhat contingent on sufficient data being available for each category. We also state that we will consider the results in relation to those of the main analysis, and that they are hypothesis-generating only.

"Subgroup analyses

Subgroup analyses will be performed to examine potential heterogeneity in treatment effect by aetiology, where sufficient data are available, as underlying cause may influence both disease progression and response to treatment. Subgroups will include:

- COVID-19
- Non-COVID-19 viral infections
- Bacterial infections
- Fungal infections
- No identified infectious cause

Furthermore, subgroup analyses will also be conducted to assess differences between drugs with distinct mechanisms of action, as this may reveal clinically meaningful variations in efficacy based on pharmacological differences. Proposed subgroups will include:

- Cytokine inhibitors stratified by cytokine (e.g., IL-1, IL-6, IL-33)
- Corticosteroids stratified by steroid (e.g., dexamethasone, hydrocortisone, prednisolone)
- Immunoglobulins
- JAK inhibitors
- G-CSF/GM-CSF
- GM-CSF inhibitors
- Plasma

A network meta-analysis (and corresponding diagram) may be presented that “lumps” the treatments in the above way, if appropriate. Finally, a subgroup analysis based on disease severity, specifically critically ill versus non-critically ill patients, will be performed, as treatment effects may differ significantly depending on baseline severity. The results of all subgroup analyses will be discussed in relation to the results of the main analysis. The subgroup analyses are secondary analyses: they are for the purpose of hypothesis generation only, and we acknowledge our use of multiple testing.”

9. Search Strategy
On Page 12, Line 20, the inclusion of a "Search term summary" appears redundant, given that full search strategies are available in the supplementary materials. I suggest removing this summary from the main text.

We acknowledge this point and have now removed the summary from the main text as suggested.

10. Risk of Bias in Individual Studies
The authors plan to use ROBINS-E for non-randomized studies. However, if randomized controlled trials are also eligible, an appropriate tool should be used for RCTs. Please clarify this and consider using multiple tools where appropriate.

We thank you for highlighting this important point. We have updated the manuscript to reflect the fact that the Cochrane RoB 2 tool will be used for randomised studies:

“For non-randomised studies the risk of bias will be assessed using the Risk Of Bias In Non-randomized Studies - of Exposure (ROBINS-E) tool and stratify studies as low risk, some concerns, high risk, very high risk, or unable to ascertain. For randomised studies the Cochrane Risk of Bias 2 tool will be used.”

11. GRADE Framework
The current reference for the GRADE framework if for pairwise meta-analyses. For network meta-analyses, I recommend adopting a framework specifically designed for this context, such as:

GRADE for NMA (Izcovich et al., BMJ, 2023), or

CINeMA (Confidence in Network Meta-Analysis; Nikolakopoulou et al., PLoS Med, 2020)

Both approaches are aligned with GRADE principles but differ in complexity and implementation.

Thank you for highlighting this important point. We have now amended the manuscript as suggested to highlight that the GRADE for NMA framework will be used.

"The quality of evidence will be assessed using the Grading of Recommendations, Assessment, Development, and Evaluations (GRADE) for network meta-analysis framework, encompassing risk of bias, imprecision, inconsistency, indirectness, and publication bias."

Alongside the above comments we have also further clarified our network meta-analysis methodology for reviewers and readers as follows:

Abstract:

"We will fit~~Jackson's model for network meta-analysis, a random-effects model that allows random inconsistency effects, using a likelihood-based approach to obtain restricted maximum-likelihood estimates~~ a random-effects network meta-analysis model that includes random effects for between-study heterogeneity and for inconsistency."

Main text:

~~"Jackson's model,~~ A random-effects network meta-analysis model ~~allowing for random inconsistency effects~~ that includes random effects for between-study heterogeneity and for inconsistency, will be used. ~~applied using a likelihood-based approach to obtain restricted maximum-likelihood estimates."~~
